# A systematic review of the effectiveness of epilepsy education programs on knowledge, attitudes, and skills among primary school learners

**DOI:** 10.3389/fneur.2024.1356920

**Published:** 2024-02-27

**Authors:** Thendo Gertie Makhado, Nombulelo Veronica Sepeng, Lufuno Makhado

**Affiliations:** ^1^Department of Advanced Nursing, University of Venda, Thohoyandou, South Africa; ^2^Department of Nursing Sciences, University of Pretoria, Pretoria, South Africa; ^3^Office of the Deputy Dean Research and Postgraduate Studies, Faculty of Health Sciences, University of Venda, Thohoyandou, South Africa

**Keywords:** epilepsy, effectiveness, learners, programs, knowledge, attitudes, skills

## Abstract

Epilepsy is a common neurological condition affecting primary school learners, often leading to misconceptions, stigma, and limited social inclusion. These misconceptions transpire because of a lack of knowledge regarding the condition and may lead to high school dropouts. This systematic review aimed to evaluate the effectiveness of epilepsy education programs on epilepsy-related knowledge and understanding, values and attitudes, and skills among primary school learners. A thorough search of electronic databases was conducted to identify relevant studies published between January 2000 and September 2023. Studies that satisfied the eligibility criteria were chosen, and two reviewers conducted data extraction independently. A narrative synthesis approach was utilised to analyse the findings. The review included 10 studies that satisfied the inclusion requirements. The epilepsy education interventions varied in content, duration, and delivery method. Findings indicated that diverse delivery methods, such as classroom-based programs, hospital-based programs, and community-centred interventions, effectively enhanced epilepsy-related knowledge and understanding, values and attitudes, and skills among primary school learners. This systematic review provides evidence that epilepsy education programs can effectively enhance epilepsy-related knowledge, understanding, values, attitudes, and skills among primary school learners. These findings support developing and implementing comprehensive guidelines for teaching epilepsy in primary schools, suggesting various delivery methods and integrating cultural values to promote optimal learning outcomes and social inclusion for learners with epilepsy.

## 1 Introduction

Epilepsy, a neurological disorder, impacts millions of individuals globally. It is characterised by recurrent seizures brought on by improper brain electrical activity (1). Epilepsy has been documented to be more prevalent among the young generation, especially between birth and the age of 12 (2, 3). Furthermore, these ages of children suffering from epilepsy are mostly learners who are found in primary schools.

Children and adolescents are among the most vulnerable populations affected by epilepsy. Certainly, epilepsy stands as a prevalent chronic neurological condition in children, with estimated occurrence rates spanning from 41 to 187 per 100,000 children (4). Epilepsy can have significant physical, psychological, and social consequences for people living with epilepsy (PLWE), including stigma, discrimination, and reduced quality of life (5, 6). Therefore, children with epilepsy often face significant challenges at school, including difficulty concentrating, memory problems, and social isolation. They may also experience stigma and discrimination from their peers and teachers due to misconceptions about the condition, leading to social isolation and school dropouts (7).

The misunderstandings surrounding epilepsy mainly stem from a lack of understanding about the condition (8). This lack of awareness also results in negative perceptions towards individuals with epilepsy (PLWE) (9). These unfavourable attitudes towards PLWE can also be observed in schools, where children spend a significant portion of their time due to teachers and peers being insufficiently informed about epilepsy (10). Consequently, it is necessary to incorporate epilepsy education into primary school curricula, allowing students to learn about epilepsy at a younger age and ensuring that teachers receive proper training. This approach could potentially reduce the stigma experienced by PLWE, both from their peers and their perceptions. Furthermore, integrating epilepsy education in schools may enhance public awareness about epilepsy as knowledge spreads from teachers and students to the broader community (3). This could ultimately foster more positive attitudes and improve seizure management skills.

Epilepsy education in schools may help diminish the stigma surrounding epilepsy and enhance the quality of life for individuals living with the condition (3). Such education may also empower people with epilepsy and their families to manage the condition more effectively and promote social and educational inclusion (3). For children with epilepsy, education programs can help them understand their condition, recognise seizure symptoms, and know how to respond to a seizure. As a result, developing and implementing practical epilepsy life skills education programs for primary school learners is critical.

The World Health Organization (WHO) emphasises education and awareness-raising as critical strategies for improving the quality of life for PLWE and reducing the societal impact of this disorder (11). The primary goal of this systematic review was to evaluate the efficacy of such educational initiatives aimed at primary school students, to increase their understanding and awareness of epilepsy, cultivating positive attitudes toward the condition, and promoting the acquisition of skills associated with identifying and reacting to seizures. We hope to gain insights into the value of incorporating epilepsy life skills education into primary school curricula through this comprehensive evaluation of their effectiveness, guided by the success of previous initiatives. Consequently, this review will enable us to formulate recommendations for developing guidelines for integrating epilepsy education within primary school settings, recognising its potential to positively impact students’ knowledge and attitudes based on the effectiveness demonstrated by previous programs.

## 2 Methods

To identify relevant studies for this systematic review, an exhaustive exploration of digital databases (PubMed, Medline, Embase, and PsycINFO) was conducted from January 2000 to September 2023. The search was limited to studies published in English. The search keywords used were “epilepsy,” “primary school learners,” teachers, “education,” and “intervention, need.” Three independent reviewers searched, and discrepancies were resolved through dialogue and agreement.

### 2.1 Eligibility criteria

The review included studies that met the following inclusion criteria:

➢ Studies published between 2000 and September 2023.
➢ The study population included primary school learners and teachers.
➢ Included an epilepsy education intervention.
➢ Compared the intervention to normal education or a control group.
➢ As outcomes, epilepsy-related knowledge and understanding, values and attitudes, and/or skills were assessed.
➢ Used a randomised controlled trial design or a quasi-experimental design.

The criteria for exclusion encompassed studies involving adults or different demographics, those lacking an epilepsy education intervention, and those not published in English. Consequently, these particular studies were not considered in the review. Initially, three reviewers independently assessed all identified studies’ titles and abstracts to determine their suitability for inclusion in the review. Subsequently, the same three reviewers acquired and independently evaluated the full texts of potentially eligible studies to validate their eligibility.

### 2.2 Selection process

**Figure 1:**
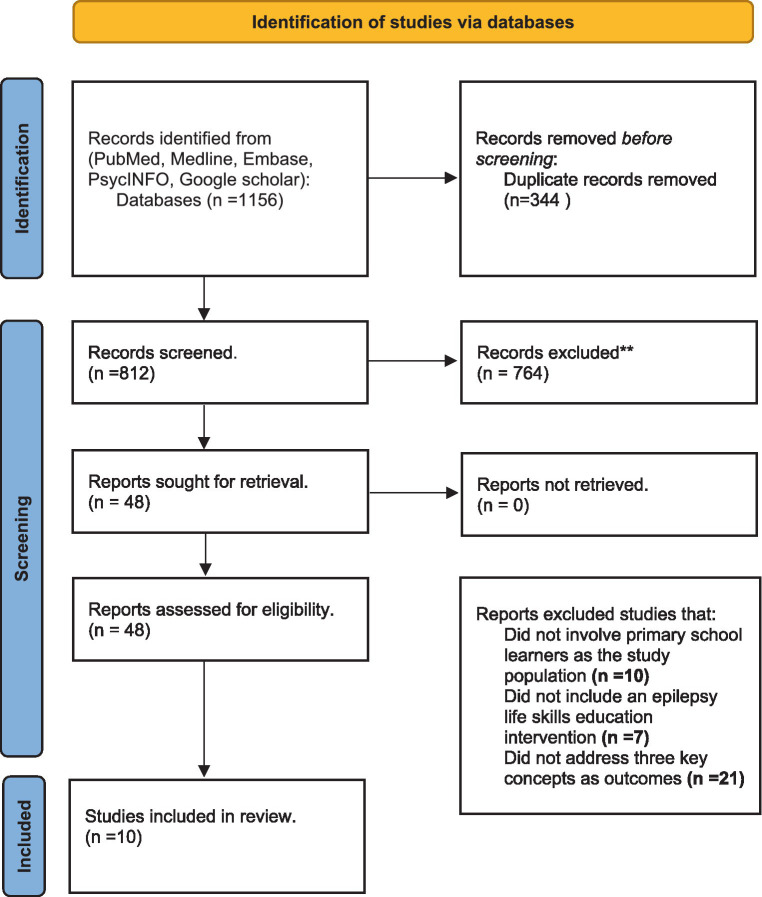
PRISMA (12).

The initial search of electronic databases yielded 1,156 potentially relevant studies. After removing duplicates, 812 studies remained. The titles and abstracts of these studies were screened independently by two reviewers, and 48 studies were identified as potentially relevant to the review. The two reviewers independently obtained and reviewed the full texts of these studies, and 10 studies were eventually included in the review. Figure 1 depicts the PRISMA flow diagram (12) depicting the study selection process, which is included in the final manuscript. Two independent reviewers extracted data from a pre-defined data extraction form. Each study yielded the following information: study design, study setting, sample size, intervention characteristics (e.g., content, duration, delivery method), outcomes assessed, and results. Disagreements in data extraction were settled through discussion and consensus.

### 2.3 Evaluation of quality

Evaluating the quality of the compiled and synthesised evidence is critical when performing a systematic review to establish recommendations. The researcher used the GRADE method in this study because it provides a transparent way to grade evidence quality for studies in the systematic review and develop recommendations (13). This approach categorises evidence into four groups: strong, moderate, weak, and extremely weak evidence (13). The quality of evidence mirrors the level of confidence the researchers have in the studies incorporated into the systematic review. High-quality evidence inspires trust, whereas low-quality evidence casts doubt on the findings.

In one study, there was a discovery of high-quality evidence concerning knowledge and comprehension of epilepsy, whereas six additional studies yielded evidence of moderate quality on the same topic (14–20). Furthermore, this provides a moderate level of confidence that educational interventions can improve primary school learners’ and teachers’ epilepsy-related knowledge and understanding. GRADE indicates that the quality of evidence for values, attitudes toward epilepsy, and skills related to seizure identification and reaction is mostly moderate to low (13–22).

Despite their moderate to low ratings, these studies should be included in the analysis. It is critical to recognise that studies with lower ratings can still provide useful information and contribute to the overall evidence base (23). Furthermore, including such studies can aid in identifying potential literature gaps or inconsistencies, which can help guide future research efforts (24).

## 3 Results

The systematic review included 10 studies meeting the set inclusion criteria. The content, duration, and delivery method of epilepsy education interventions differed. The interventions contained information about epilepsy, seizure recognition and management, medication management, first aid, and coping strategies. The studies employed a variety of measures to assess epilepsy-related knowledge and understanding, values and attitudes, and skills. The measures included questionnaires, interviews, and observations (14, 16–22, 25). Some studies used standardized measures, such as the Epilepsy Knowledge and Attitudes Scale (EKAS) or the Seizure Recognition and First Aid Questionnaire (SRFAQ), while others used study-specific measures (15).

### 3.1 Knowledge and attitude

All studies found that the epilepsy life skills education interventions improved epilepsy-related knowledge and understanding compared to normal education or a control group (14–22, 25). The interventions were found to be effective in improving knowledge and understanding of epilepsy in terms of its causes, symptoms, and management, as well as reducing misconceptions and improving knowledge of first aid for seizures (12, 14, 15).

### 3.2 Values and attitudes

In addition, most studies found that the interventions also improved values and attitudes towards epilepsy (14, 16–18, 20, 22, 25). Participants who received the epilepsy education interventions had higher positive attitudes towards PLWE, were less likely to hold negative stereotypes, and were more likely to support the inclusion of PLWE in social and educational activities (14, 16–18, 20, 22, 25).

### 3.3 Skills of recognizing and managing seizures

Several studies also found that the interventions improved skills related to epilepsy, such as recognizing seizure symptoms and knowing how to respond to a seizure (14, 16, 17, 19). Participants who received the interventions were better able to recognise different types of seizures, knew what to do in case of a seizure, and were more likely to report that they would provide appropriate first aid for someone experiencing a seizure (14, 16, 17, 19).

Overall, the studies analyzed in this systematic review offer compelling evidence that epilepsy life skills education programs can help primary school students learn more about epilepsy, develop positive attitudes and values toward the condition, and develop skills related to recognizing and handling seizures. The traits of the included studies are displayed in Table 1.

**Table 1:** Studies included and their characteristics.

Authors	Aim of the study	Population	Study design	Main findings
(21)	This study aims to determine the effectiveness of an instructional program on elementary school teacher’s knowledge concerning epilepsy	Elementary teachers	A quasi-experimental design with the application of a pre-posttest approach	The study’s findings showed that many of the study sample’s elementary school learners in Baghdad’s Al-Rusafa third education directorate lacked sufficient knowledge about epilepsy. However, the Al-Rusafa Third Education Directorate in Baghdad City teachers’ understanding of epilepsy improved after implementing the study group’s instructional program. This demonstrates how well the program works to improve teachers’ understanding of epilepsy
(18)	To assess if an epilepsy education program (intervention) enhances the knowledge of epilepsy and fosters positive attitudes among Grade 5 students (aged 9–11)	Grade 5 students ages 9–11	Stratified cluster randomised controlled trial	Unlike the control group, the intervention group exhibited a notable rise in their epilepsy knowledge and positive attitudes one month after participating in the epilepsy education program
(22)	To evaluate the relative effectiveness of an educational animated video and educational drama in enhancing epilepsy knowledge and reducing epilepsy-related stigma among children aged 9 to 11 years	Primary school learners	Controlled experimental design	This study showed that educational videos and educational dramas effectively increased knowledge about epilepsy and decreased stigma related to epilepsy among primary students aged 9 to 11. Both techniques enhanced epilepsy knowledge and more favorable attitudes toward learners with the condition
(17)	This study aimed to explore the existing knowledge and practices teachers employ in handling epileptic seizures while also assessing the impact of an epilepsy intervention educational package on enhancing teachers’ knowledge and practices related to epilepsy management	Teachers	Pretest-post-test design	The intervention positively impacted, reducing misconceptions and increasing knowledge, including recognising warning signs and appropriate responses to seizures among teachers. The intervention also had the impact in instilling positive attitudes towards people living with epilepsy and the ability to recognise and manage seizures
(16)	To examine the effect of epilepsy health education on knowledge, attitude and first aid management of epilepsy on teachers in Nigeria	Trainee teachers	Pretest-post-test design	Providing health education on epilepsy can enhance trainee teachers’ understanding of epilepsy, improve their attitudes towards it, and enable them to correctly administer first aid when necessary. This underscores the importance of integrating a specialized epilepsy intervention program into the training curriculum for teachers
(20)	The objective was to evaluate how an interactive single-day educational workshop on epilepsy affected the existing levels of knowledge, attitudes, and practices related to epilepsy among school teachers	School Teachers	Pretest-post-test design	The educational intervention yielded outcomes regarding knowledge, practices, and attitudes related to epilepsy. However, it also resulted in some unanticipated adverse effects. A solitary workshop may not suffice to deliver precise information about epilepsy
(19)	The study’s objective is to assess participants’ understanding and perceptions of epilepsy, as well as to determine whether a concentrated and targeted educational program could lead to enhancements in both knowledge and attitudes	Primary school teachers	Pretest-post-test design	Education effectively enhances the epilepsy-related knowledge of primary school teachers, enabling them to manage better a child experiencing a seizure. However, it does not significantly alter the perception that epilepsy leads to social disadvantage. Education is more likely to mitigate ignorance rather than prejudice
(15)	To assess the efficacy of two interventions to diminish stigma associated with epilepsy in children aged 9 to 11 years	Primary school pupils aged 9–11	Pretest-post-test design	The findings demonstrated that providing epilepsy education to primary school learners can effectively diminish the stigma associated with epilepsy
(25)	The objectives of this study involve investigating how a psycho-educational program influences teachers’ perceptions and attitudes concerning epilepsy in children	Primary school teachers	Quasi-experimental self-controlled design	The finding of this study has revealed that before the implementation of the psycho-educational program, primary school teachers had negative attitudes and beliefs towards epilepsy
(14)	To assess the immediate effects of epilepsy training in Lebanese public and private schools. The evaluation was conducted both before and right after the intervention	Three groups of teachers and counsellors	Pretest-post-test design	The results of this study demonstrated that, following the intervention, the group comprising teachers and counsellors exhibited enhanced knowledge and improved comprehension of epilepsy. Additionally, their attitudes towards individuals living with epilepsy became more positive, and they acquired the skills necessary for providing first aid during seizures. Furthermore, they recognised the importance of referring learners experiencing seizures lasting longer than 5 min

## 4 Discussion

The results of this systematic review showed that epilepsy education programs are successful in enhancing primary school learner’s epilepsy-related knowledge and understanding of epilepsy, values and attitudes, and skills related to identifying and reacting to seizures (14–22, 25). In order to lessen stigma and discrimination against PLWE, it is essential to increase knowledge and understanding of the condition. Education programs can assist in dispelling myths and misconceptions about epilepsy by providing accurate information about the causes, symptoms, and management of the condition. This can lessen social isolation among those with epilepsy by fostering a more accepting and supportive environment. The results of this study suggest that epilepsy education programs can successfully increase learner’s knowledge and understanding of epilepsy, which can be advantageous for PLWE and their families in the long run.

In addition to improving knowledge and understanding, the epilepsy education programs included in this review were also effective in improving values and attitudes towards epilepsy (14, 16–18, 20, 22, 25). By promoting positive attitudes towards PLWE and reducing negative stereotypes, these programs can help create a more inclusive and accepting environment for PLWE. This is especially important for children with epilepsy, who may experience social exclusion and discrimination at school due to misconceptions. The findings of this review suggest that epilepsy education programs can help reduce stigma and promote social inclusion for primary school learners with epilepsy (15, 22).

Finally, several studies found that seizure recognition and response skills were also improved by the epilepsy life skills education programs (14, 16, 17, 19). These programs can help lower the risk of injury and enhance the quality of life for PLWE by educating learners about the various types of seizures and equipping them with the abilities and knowledge to react appropriately. According to the results of this review, integrating epilepsy education programs into primary school curricula can help to improve the security and wellbeing of learners with epilepsy.

## 5 Conclusion

The results of this systematic review offer compelling evidence that epilepsy life skills education programs successfully enhance primary school students’ epilepsy-related knowledge and understanding, values and attitudes, and skills related to recognising and handling seizures. To lessen stigma and enhance the quality of life for those who have epilepsy, healthcare professionals, educators, and policymakers should think about including epilepsy education programs in primary school curricula. Additional research is required to determine the interventions’ most compelling content, duration, and delivery strategy. The systematic review acknowledges limitations, particularly the heterogeneity in the design of the educational programs and the methods used to evaluate their outcomes. This diversity in program content, duration, and delivery method, as well as the variety of assessment measures used (like questionnaires, interviews, and observations), made it challenging to conduct a meta-analysis. Consequently, there was a limitation in summarizing the results quantitatively. This heterogeneity reflects the complexity and varied contexts of epilepsy education programs, impacting the ability to draw generalized conclusions from the study.

## Data availability statement

The original contributions presented in the study are included in the article/supplementary materials, further inquiries can be directed to the corresponding author.

## Author contributions

TM: Conceptualization, Data curation, Formal analysis, Funding acquisition, Investigation, Methodology, Resources, Validation, Visualization, Writing – original draft, Writing – review & editing. NS: Data curation, Formal analysis, Writing – review & editing. LM: Data curation, Formal analysis, Supervision, Writing – review & editing.
